# Traité Internationale de Psychologie Pathologique

**Published:** 1911-12

**Authors:** 


### Reviews of Books.

**Traité Internationale de Psychologie Pathologique.** Edited by Dr. A. MARIE and Collaborators. Tome ii. Pp. xxiii, 1000. Paris: Félix Alcan. 1911.

This the second volume well sustains the high level reached by the first. The subjects dealt with are of much interest to the practical alienist, including neurasthenia and the psycho-neuroses, syphilis and various organic affections in relation to mental disorders, general paralysis, various forms of dementia, the degenerescent, chronic and systematised psychoses, precocious dementia, cyclothymic, toxic and confusional states. In such a work as this, where the contributors are numerous, a certain loss of serial continuity and some overlapping of subjects is inevitable, as is also some discrepancy of opinions. Moreover, the several papers complete in themselves are often too elaborate disquisitions for the student, and needlessly retrospective for the advanced reader. Such defects are inseparable from these joint-stock productions, but capable editing has reduced them so far as is possible.

There are many eager investigators into morbid psychology, and we are at present liable to numerous temporary schemes of classification and nomenclature, which in their transitional state occasion no small perplexity to the student. Thus the elaboration of such syndromes as dementia precox, the paranoical and allied states, the various forms of chronic systematised delirium, toxic states of confusional psychosis has quite doubled the space required for a treatise on insanity but a few years ago. In this work all the debatable points in connection with these are fully discussed, and it is apparent that even now much will remain debatable for the present. The morbid brain is capable of producing a great number of kaleidoscopic variations in its symptomatology, and it will be found necessary in time to eliminate many superfluous combinations which are at present exploited as constant syndromes. This filtration process is already at work—in dementia precox, for example. A good deal is written as to the relations between paranoical and paranoidal delirium, between the latter and chronic systematised insanity; but these are complex matters and much in the stage of academic discussion only.

Dr. Marie's article on General Paresis is all-embracing. He emphasises the manifold nature, perhaps origin, of this affection. In so far as the Wassermann positive reaction represents syphilis, so do his and Levaditi's findings support the parasyphilitic origin of general paresis. Recent observers have not supported these large percentages, nor do they admit the diagnosis unless a positive cerebro-spinal fluid reaction is obtained No confirmation, but the reverse, is given to Robertson's claims as regards the specific action of the bacillus paralyticans in general paresis. Marie lays stress on the therapeutic importance of Wassermann reaction in deciding the transition of syphilis to parasyphilis in presumably syphilitic general paralytics By comparing parallel reactions of serum and cerebro-spinal fluid this stage he thinks may be estimated and the propriety or not of specific mercurial treatment resolved upon. The other sections of the work do not call for special comment; they are all good, and of a part with the general production as regards printing and illustrations—we cannot add binding.

